# A phase 2 double-blind placebo-controlled 24-week treatment clinical study of the p38 alpha kinase inhibitor neflamapimod in mild Alzheimer’s disease

**DOI:** 10.1186/s13195-021-00843-2

**Published:** 2021-05-27

**Authors:** Niels D. Prins, John E. Harrison, Hui-May Chu, Kelly Blackburn, John J. Alam, Philip Scheltens, for the REVERSE-SD Study Investigators, Arnold, Coskinas, Gonzales, Joseph, Khan, McConnehey, Paricio, Taylor, Zarate Rowell, Groom, Summers, White, Thein, Bolouri, Schwartz, Vasquez, Asher, Ball, Munthali, Kabir, Langford, Komuravelli, Lynch, MacSweeney, Pearson, Faulkner, Miller, Underwood, Prins, Dautzenberg, Van Norden, Pazdera, Bar, Dvacka, Valis, Votypkova, Areovimata, Justesen, Schmidt

**Affiliations:** 1 Alzheimer Center, Department of Neurology, Amsterdam UMC, Amsterdam, The Netherlands; 2 Brain Research Center, Amsterdam, The Netherlands; 3 Institute of Psychiatry, Psychology & Neuroscience, King’s College London, London, UK; 4 Metis Cognition Ltd., Wiltshire, UK; 5 Anoixis Corporation, Natick, MA USA; 6 EIP Pharma, Inc., Boston, MA USA

**Keywords:** p38 MAPK, Alzheimer’s, Episodic memory, Clinical trial, CSF biomarkers, Synaptic dysfunction

## Abstract

**Background:**

In preclinical studies, p38⍺ kinase is implicated in Alzheimer’s disease (AD) pathogenesis. In animal models, it mediates impaired synaptic dysfunction in the hippocampus, causing memory deficits, and is involved in amyloid-beta (Aβ) production and tau pathology.

**Methods:**

The REVERSE-SD (synaptic dysfunction) study was a multi-center phase 2, randomized, double-blind, placebo-controlled trial of the p38⍺ kinase inhibitor neflamapimod; conducted December 29, 2017, to June 17, 2019; 464 participants screened, and 161 randomized to either 40 mg neflamapimod (78 study participants) or matching placebo (83 study participants), orally twice daily for 24 weeks. Study participants are as follows: CSF AD-biomarker confirmed, Clinical Dementia Rating (CDR)-global score 0.5 or 1.0, CDR-memory score ≥0.5, and Mini-Mental State Examination (MMSE) 20–28. The primary endpoint was the improvement in episodic memory, assessed by combined change in *Z*-scores of Hopkins Verbal Learning Test-Revised (HVLT-R) Total and Delayed Recall. Secondary endpoints included change in Wechsler Memory Scale-IV (WMS) Immediate and Delayed Recall composites, CDR-SB, MMSE, and CSF biomarkers [total and phosphorylated tau (T-tau and p-tau_181_), Aβ_1-40_, Aβ_1-42_, neurogranin, and neurofilament light chain].

**Results:**

At randomization, the mean age is 72, 50% female, 77% with CDR-global score 0.5, and mean MMSE score 23.8. The incidence of discontinuation for adverse events and serious adverse events (all considered unrelated) was 3% each. No significant differences between treatment groups were observed in the primary or secondary clinical endpoints. Significantly reduced CSF levels with neflamapimod treatment, relative to placebo, were evident for T-tau [difference (95% CI): −18.8 (−35.8, −1.8); *P*=0.031] and p-tau_181_ [−2.0 (−3.6, −0.5); *P*=0.012], with a trend for neurogranin [−21.0 (−43.6, 1.6); *P*=0.068]. In pre-specified pharmacokinetic-pharmacodynamic (PK-PD) analyses, subjects in the highest quartile of trough plasma neflamapimod levels demonstrated positive trends, compared with placebo, in HLVT-R and WMS.

**Conclusions and relevance:**

A 24-week treatment with 40 mg neflamapimod twice daily did not improve episodic memory in patients with mild AD. However, neflamapimod treatment lowered CSF biomarkers of synaptic dysfunction. Combined with PK–PD findings, the results indicate that a longer duration study of neflamapimod at a higher dose level to assess effects on AD progression is warranted.

**Trial registration:**

ClinicalTrials.gov identifier: NCT03402659. Registered on January 18, 2018

**Supplementary Information:**

The online version contains supplementary material available at 10.1186/s13195-021-00843-2.

## Background

Synaptic dysfunction has emerged as a therapeutic target for Alzheimer’s disease (AD), particularly in the early stages of disease [1–3]. In animal models of neurodegenerative disease intervention, approaches that target stress-activated intracellular pathways have shown the ability to both restore function and prevent further neuronal loss, if initiated early in the disease process, after neuronal loss has begun but before it has become widespread [4]. The alpha isoform of p38 mitogen-activated-protein kinase (p38α) is an intracellular protein that is considered to be a leading therapeutic target for treating synaptic dysfunction in AD [5–9]. Expression of p38α in the neuron is associated with the formation of pathological amyloid-beta (Aβ), inflammation, and tau-induced synaptic dysfunction [7, 10–17]. Studies in several distinct animal models, each driven either by Aβ, inflammation, or tau, showed that spatial learning deficits are reversed with small molecule inhibitors of p38α kinase activity [18–20], providing direct evidence that p38α inhibition could be beneficial in AD. Moreover, genetic reduction of neuronal p38α levels in amyloid-precursor-protein overexpressing transgenic mice improved synaptic transmission, decreased memory loss, and reduced amyloid pathology [21, 22]. Genetically, suppressing p38α expression in mice also prevented age-related decline in hippocampal function [23, 24]. This is consistent with a human genetic linkage study identifying the p38α pathway as a modifier of age-related decline in episodic memory [25] and mechanistic studies showing that p38α modulates memory formation [26].

The investigational drug neflamapimod is an oral, brain-penetrant, selective p38α inhibitor that reversed functional deficits in performance in the Morris Water Maze in aged rats [18]. Two small (25 patients in total) phase 2a clinical studies of neflamapimod in early AD, without a placebo control, showed good tolerability, adequate drug concentrations in CSF, and trends for improving episodic memory [27, 28]. To further evaluate its effects on synaptic dysfunction, as evaluated by tests of episodic memory and CSF biomarkers, we performed a placebo-controlled clinical trial, named REVERSE-SD (synaptic dysfunction), of this p38α kinase inhibitor in patients with mild AD. The primary objective of the trial was to determine whether 24 weeks of treatment with neflamapimod improves episodic memory (a direct measure of synaptic dysfunction in the hippocampus) and decreases levels of CSF biomarkers considered to be associated with synaptic dysfunction in AD.

## Methods

### Participants and study design

This was a multi-center phase 2, randomized, parallel-group, double-blind placebo-controlled trial conducted in 38 centers in the US (16 centers, 45% of participants), the UK (11, 32.5%), the Netherlands (3, 12.5%), Czech Republic (5, 6%), and Denmark (3, 4%). One hundred sixty-one patients were randomized, of whom 78 received neflamapimod and 83 placebos. The first participant was enrolled on December 29, 2017, and the last visit occurred on June 17, 2019. The study was conducted in accordance with Good Clinical Practice guidelines [29] and the Declaration of Helsinki [30]. Applicable local/central ethics committee or IRB approvals were obtained, and all participants provided written informed consent.

During the treatment period, participants attended study center visits on week 3, week 6, week 12, week 18, and week 24. Participants took study drug twice daily with a meal for 24 weeks. The main clinical outcome measures, the Hopkins Verbal Learning Test-Revised (HVLT-R) and the Wechsler Memory Scale-IV (WMS), were conducted on day 1 (baseline) and on weeks 6, 12, and 24. Lumbar puncture to collect CSF was conducted during the screening period and repeated at week 24.

Participants eligible for randomization were 55 to 85 years of age; had a Clinical Dementia Rating Scale (CDR) Global Score of 0.5 or 1.0, a CDR memory sub-score ≥0.5, CSF Aβ_42_ <1000 pg/mL, and CSF p-tau_181_/Aβ_42_ ratio >0.024 by Roche Elecsys® assay; and Mini-Mental State Examination (MMSE) score of 20 to 28, inclusive. Participants on a single-drug AD therapy (cholinesterase inhibitors or memantine, dual therapy excluded) could remain on that background AD therapy if on a stable dose for ≥2 months and the dose was not changed during the trial. Randomization was conducted by a central Interactive Web Response System and stratified by CDR global score (0.5/1.0) and use of background AD therapy (yes/no).

All participants received their first dose of the study drug at the clinical site. Thereafter, participants were instructed to take a study drug were taken within 30 min following a meal or snack (i.e., breakfast and dinner) no less than 8 h apart and at approximately the same times each day throughout the study. The count of returned capsules at each visit was reviewed, and any apparent discrepancies between the quantity of capsules returned and the number expected based on the dosing schedule were discussed with the subject to ensure an understanding of dosing instructions. Participants were also provided with identification cards on which they recorded the date and time of study drug administration prior to each scheduled study center visit.

### Primary outcome measure

The HVLT-R [31] is a word list verbal learning test that evaluates episodic learning and memory. Each of the six alternative forms of the test consists of a 12-item word list, composed of four words from each of the three semantic categories. Total Recall score (scored 0–36, the sum of three immediate recall trials) and Delayed Recall score (scored 0–12; single recall, 20 to 25 min after initial trials) were assessed. To minimize learning effects, form A was utilized on day 1 (baseline), form B on week 6, form C on week 12, and form D on week 24. We calculated *Z*-scores for Total Recall and Delayed Recall scores on the HVLT-R.

### Secondary outcome measures

#### Cognitive measures

Participants were also assessed using episodic memory components of the WMS [32]. Performance was assessed using composite measures of both immediate and delayed recall. The WMS Immediate Recall composite score at each testing session consisted of the sum of the scores for Logical Memory (LM) I, Verbal Paired Associates (VPA) I, and Visual Reproduction (VR) I. The WMS Delayed Recall composite score consisted of the sum of LM II, VPA II, and VR II. To obtain data for designing future studies of longer duration, CDR sum-of-boxes (CDR-SB) and MMSE scores were also assessed.

#### Cerebrospinal fluid measures

Kits for CSF sample collection were provided to the sites and aliquots for endpoint biomarker analyses were stored at −80°C until the end of the study. CSF protein levels of total tau (T-tau), phosphorylated tau (p-tau_181_), Aβ_40_, Aβ_42_, neurogranin, and neurofilament light chain (NfL) were determined with commercially available enzyme-linked immunosorbent assay kits in the laboratory of Charlotte Teunissen at Amsterdam UMC, Amsterdam, NL (Roche Elecsys®: T-tau, P-tau_181_, Aβ_42_; Euroimmun: Aβ_40_; ADx Neuroscience: neurogranin; Uman Diagnostics: NfL).

### Pharmacokinetics

For trough plasma drug concentration determinations, on day 21, participants were instructed and called the day before to not take their morning dose of study drug at home; instead, that dose was administered at the study center and blood samples collected for pharmacokinetic (PK) testing immediately prior to study drug administration. In addition, that the participant had taken their second dose of study drug the evening before was confirmed and the time of that dose was recorded. Drug concentrations were determined utilizing a validated LC–MS/MS assay [33].

### Statistical analysis

The efficacy population included the 160 participants who received their first dose of study drug (in all cases at the clinical site) and had at least one post-dose efficacy assessment. The primary efficacy endpoint was the combined change in *Z*-score of Total Recall and Delayed Recall on the HVLT-R in neflamapimod-treated subjects, compared with placebo recipients, at week 24. We analyzed the relationship between neflamapimod use and change in the primary endpoint, as well as in WMS and CDR-SB scores, using Mixed Model for Repeated Measures (MMRM) with fixed effects for treatment, background AD therapy, CDR-Global Score of 0.5 versus 1.0, scheduled visit (nominal) and scheduled visit by treatment interaction, random effect for subject and baseline *Z*-score as a covariate. Least-square means (LSM) and 2-sided 95% confidence intervals (CI) are provided for treatment group differences and estimated endpoint values by visit. The original sample size of 76 patients per arm provided 90% power to detect an effect size (ES) of 0.53 and 80% power to detect an ES of 0.46 on the primary endpoint.

Changes in CSF biomarkers and MMSE scores were compared using an ANCOVA with treatment group, background AD therapy, and baseline CDR-Global Score as main effects and the baseline assessment as the covariate. The results of the ANCOVA are summarized using the treatment group LSMs, the difference between the treatment group LSMs, the 95% confidence interval for the treatment group difference, and the *p* value.

## Results

### Patients, demographics, and enrollment

**Fig. 1:**
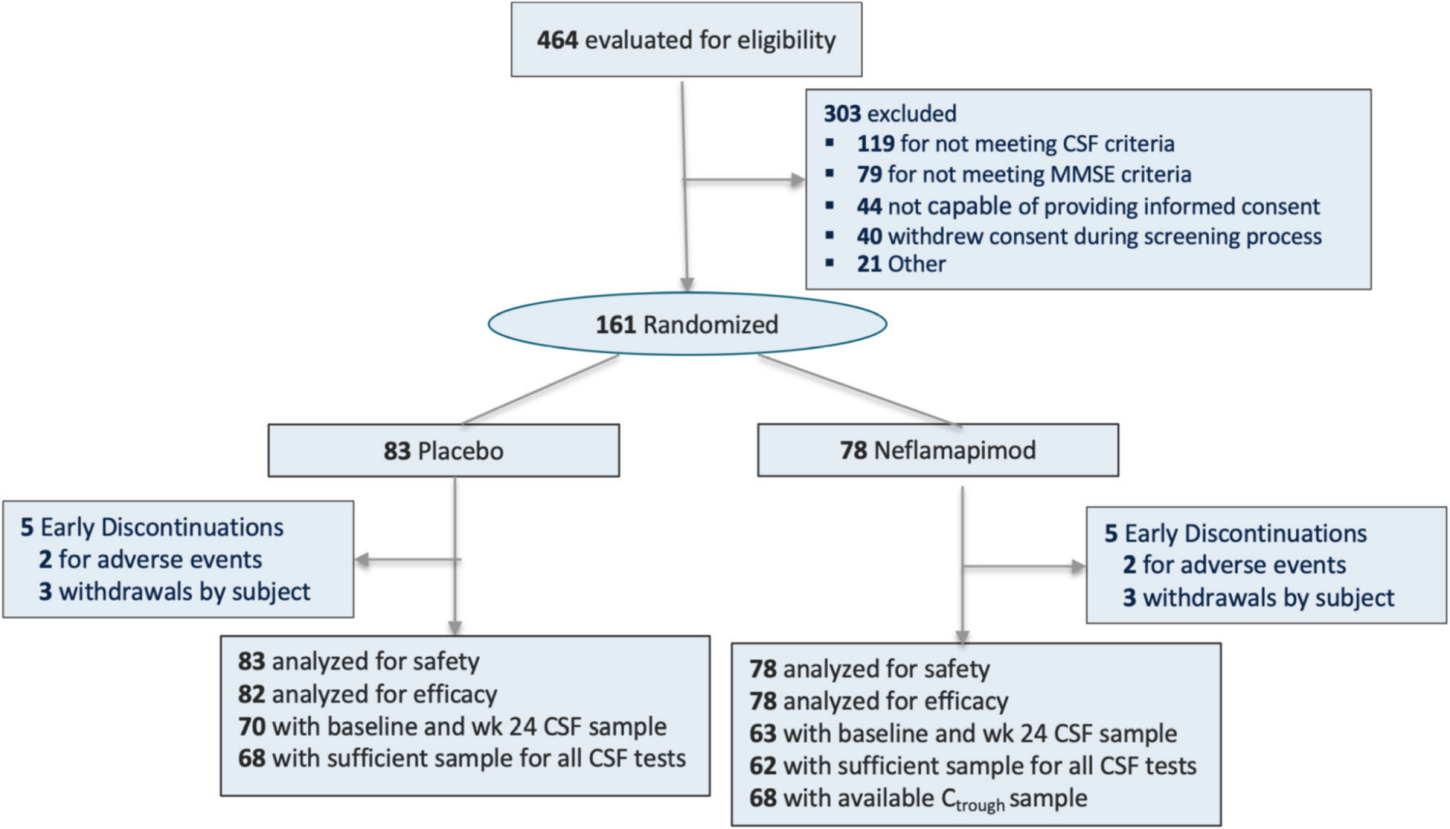
CONSORT flow diagram

Four hundred sixty-four patients entered screening and 161 were randomized; 83 to the placebo group and 78 to the neflamapimod 40 mg group (Fig. 1). Among randomized patients, 50% were males and 50% females. The mean (SD) age of subjects was 72 (6.8) years. Most subjects were white (156 subjects, 97%). One hundred twenty-five (78%) patients had a global CDR-Global score of 0.5 (mild cognitive impairment) and 36 (22%) had a score of 1 (mild dementia). The mean MMSE score was 23.8 (2.48). All patients randomized received at least one dose of study drug and were included in assessments of safety. All but one placebo recipient had at least one on-treatment efficacy assessment, and so 82 placebo recipients and 78 neflamapimod recipients were included in the efficacy analysis population. Based on counts of returned capsules, 91.8% of placebo-recipients and 93.6% of neflamapimod-recipients received greater than 90% of their planned study doses.

Table 1 gives the baseline characteristics by treatment group and whether the patients were receiving background AD therapy, a stratification variable in the randomization. The patients receiving background AD therapy had significantly more advanced disease while, within each stratum (with or without background AD therapy), there were no significant differences between neflamapimod and placebo recipients for any of the clinical and CSF measures at baseline. Of neflamapimod and placebo recipients, 59% and 61%, respectively, received background AD therapy (85% taking a cholinesterase inhibitor and 15% memantine).

**Table 1:** Baseline disease characteristics by background AD therapy

	Placebo		Neflamapimod	
	AD therapy (*n* = 51)	No AD therapy (*n* = 32)	AD therapy (*n* = 46)	No AD therapy (*n* = 32)
Age	73 (7.8)	72.1 (5.9)	72.2 (6.8)	68.9 (5.6)
Gender (% female)	45.1	62.5	47.8	46.9
ApoE4 (% positive)	66.7	71.9	67.4	78.1
HVLT-R Total Recall	13.4 (4.5)	18.3 (4.9)	14.8 (5.1)	18.6 (6.9)
HVLT-R Delayed Recall	1.8 (2.1)	4.0 (3.3)	2.2 (2.4)	4.7 (4.0)
WMS Immediate & Delayed Recall	58.1 (21.8)	84.2 (34.1)	61.7 (25.0)	92.6 (42.0)
CDR sum of boxes	3.7 (1.6)	2.4 (1.5)	3.4 (1.4)	2.6 (1.0)
MMSE	22.7 (2.4)	25.2 (2.7)	22.9 (2.5)	25.1 (2.3)
CSF Aβ_40_	10583 (3340)	10783 (3162)	11099 (2926)	11259 (3465)
CSF Aβ_42_	545 (155)	562 (194)	556 (138)	602 (196)
CSF p-tau_181_	35.5 (14.2)	30.9 (11.0)	37.6 (15.1)	33.4 (14.4)
CSF total tau	360 (126)	316 (97)	378 (132)	337 (116)
CSF neurogranin	477 (238)	427 (160)	510 (224)	469 (161)
CSF NfL	1456 (640)	1230 (694)	1586 (760)	1257 (530)

Mean (SD), except where percentage is shown. CSF levels are shown as pg/mL

### Full efficacy population analyses

Table 2 gives the results of clinical endpoint analyses of the full efficacy population (Table 2). The primary endpoint was not met, with no significant difference between neflamapimod treatment and placebo in the change from baseline to week 24 in the combined *Z*-score of Total Recall and Delayed Recall in the HVLT-R. Similarly, no significant differences between neflamapimod and placebo were seen over the same time period in secondary clinical efficacy measures, including WMS Immediate and Delayed Recall composite scores, CDR-SB scores, and MMSE scores.

**Table 2:** Changes of clinical endpoints from baseline to week 24

Endpoint	Placebo (***n*** = 82)	Neflamapimod (***n*** = 78)	Difference in change (95% CI)	***P*** value
HVLT-R combined *Z*-score Total and Delayed Recall	−0.13 (−0.27, 0.01)	−0.17 (−0.38, 0.05)	−0.03 (-0.23, 0.16)	NS
WMS Immediate & Delayed Recall	16.6 (11.1, 22.1)	16.0 (10.5, 21.5)	-0.6 (−6.0, 4.8))	NS
CDR-SB	1.0 (0.5, 1.5)	1.1 (0.6, 1.7)	0.1 (−0.4, 0.6)	NS
MMSE	−0.5 (−1.3, 0.3)	−0.8 (−1.7, 0.1)	−0.3 (−1.0, 0.5)	NS

Except for MMSE, results shown derived from mixed model for repeated measures (MMRM) analysis of change from baseline to week 24. MMSE results from ANCOVA of change from baseline to week 24. Least square means (95% confidence interval) from models are shown

**Fig. 2:**
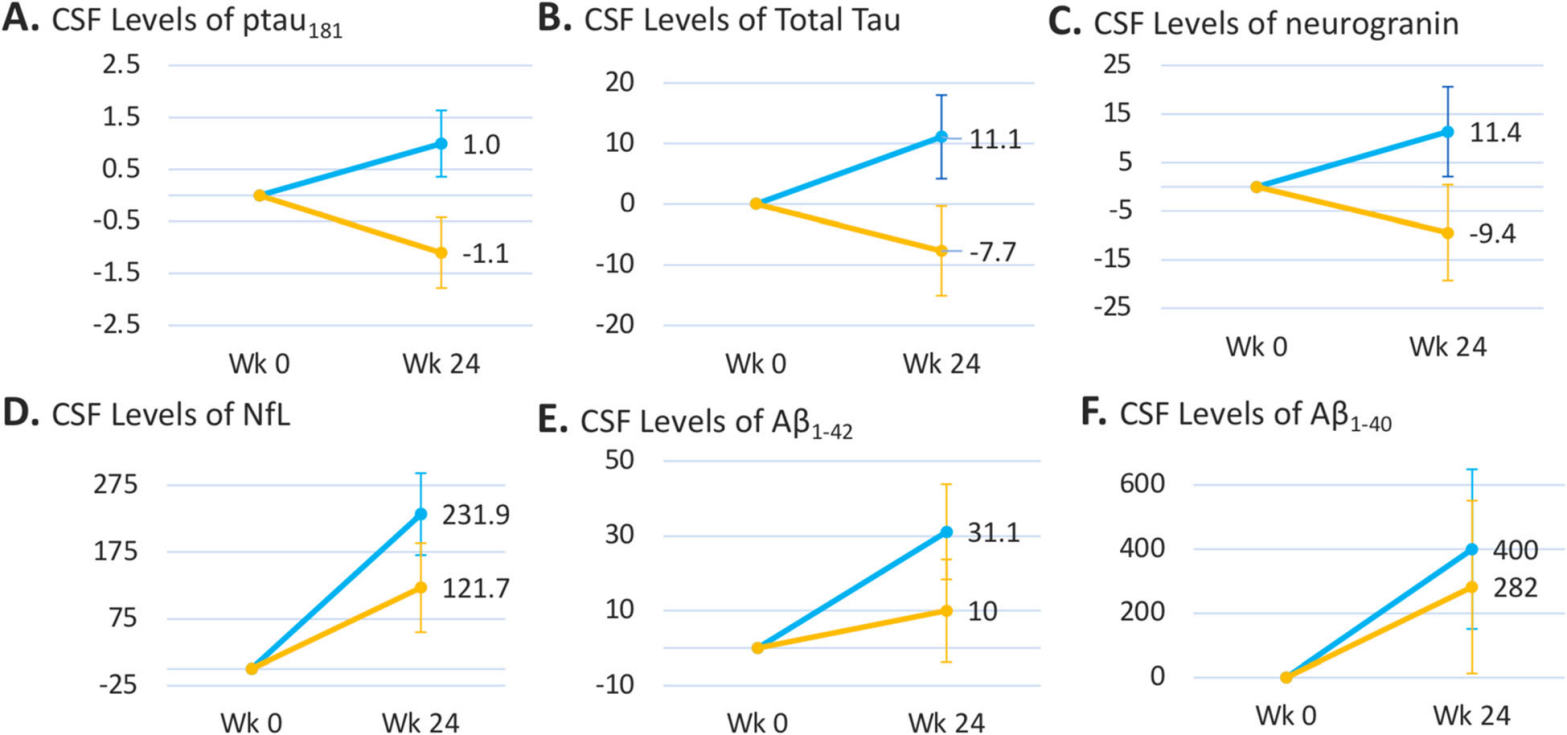
Results of CSF biomarkers of neurodegeneration and synaptic dysfunction. Mean (s.e.m.) absolute (pg/mL) change from baseline to week 24 CSF sampling is shown. The difference between neflamapimod treatment and placebo was significant for T-tau [difference (95% CI): −18.8 (−35.8, −1.8); *P*=0.031] and p-tau_181_ [−2.0 (−3.6, −0.5); *P*=0.012], with a trend for neurogranin [−21.0 (−43.6, 1.6); *P*=0.068.  N=68 for placebo and N=62 for neflamapimod for A, B, E and F.  N=70 for placebo and N=63 for neflmapimod for C and D.]

Figure 2 shows CSF biomarker results in the full efficacy population. Improvements, based on decreased levels of the disease biomarkers CSF T-tau [difference (95% CI): −18.8 (−35.8, −1.8); *P*=0.031] and CSF p-tau_181_ [−2.0 (−3.6, −0.5); *P*=0.012], were seen over the 24-week neflamapimod treatment period, relative to placebo. Furthermore, CSF neurogranin levels showed a trend [−21.0 (−43.6, 1.6) *P*=0.068] towards improvement with neflamapimod treatment, relative to placebo administration. Of note, the standard deviation relative to mean baseline level was greater for neurogranin than for either T-tau or p-tau, providing a potential explanation for the higher *p* value despite a similar proportionate difference for the CSF neurogranin comparison between neflamapimod and placebo groups.

### PK–PD analyses

**Fig. 3:**
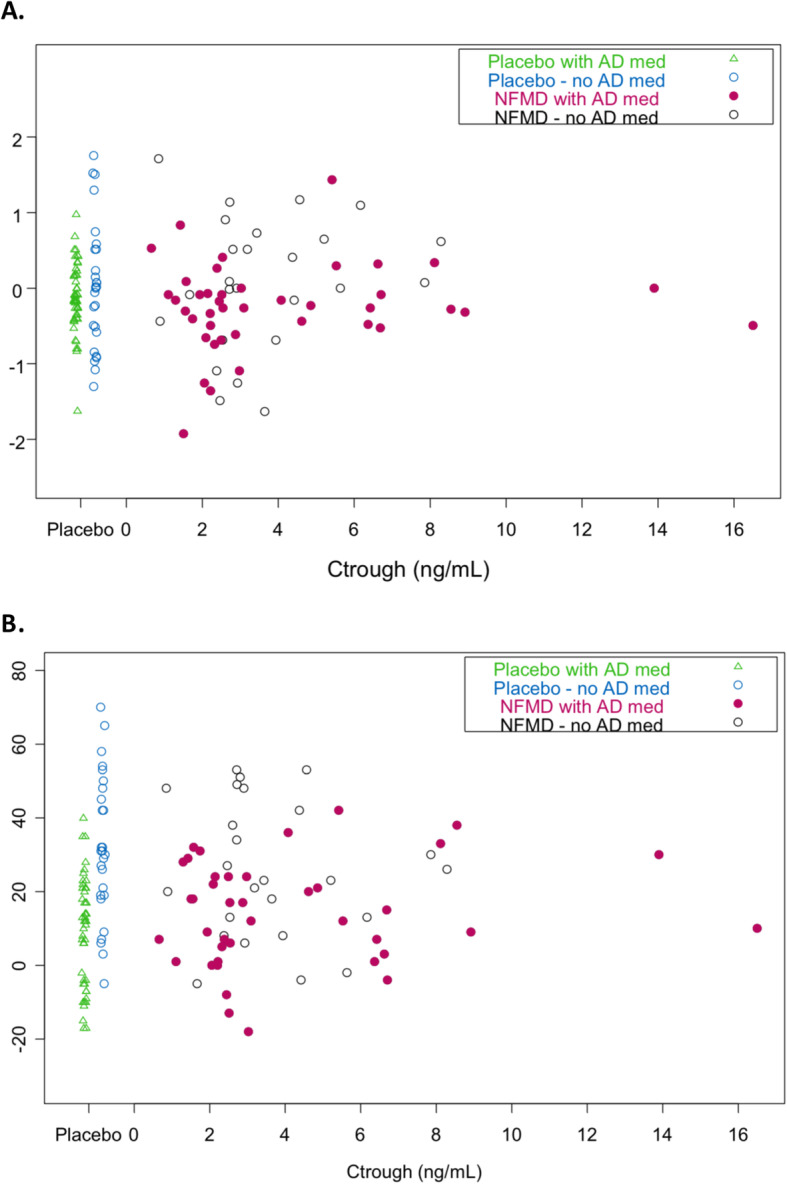
Relationship between C_trough_ and episodic memory measures. Plasma trough drug concentration in neflamapimod versus change from baseline to week 24. **a** HVLT-R combined total and delayed recall *Z*-score (i.e., primary endpoint), and **b** WMS combined immediate and delayed recall composite is shown in circles (open for treatment-naïve subjects, closed for those on background AD therapy). For comparison, placebo subjects are shown in triangles (open for treatment-naïve subjects, closed for those on background AD therapy) on the left side of the figure

All participants randomized to neflamapimod had quantifiable plasma drug concentrations at their day 21 pre-dose plasma sample (C_trough_) determination, indicating that participants had received their dose of study drug the evening before. Figure 3a illustrates the relationship between C_trough_ and the primary endpoint. In the placebo group, subjects on background AD therapy had a median *Z*-score decline of approximately 0.15 over the 24 weeks in the trial, while those not on background AD therapy had a median of no change over the 24 weeks. There was high inter-subject variability (standard deviation for change in *Z*-score of 0.6 for the placebo group as a whole). In the neflamapimod-treated subjects, examination of the outcomes by C_trough_ revealed an apparent threshold effect in the change from baseline to week 24 in the primary endpoint. Thus, subjects with C_trough_ levels lower than approximately 4 ng/mL had a distribution similar to that in the placebo group while, in those with C_trough_ levels greater than 4 ng/mL, the distribution was shifted upwards with fewer subjects showing a decline, i.e., less disease progression. In a pre-specified analysis, the primary endpoint outcome in the neflamapimod subjects in the highest quartile of C_trough_ (cut-off = 5.4 ng/mL) was compared to placebo recipients and the subjects in the lower three quartiles of neflamapimod C_trough_. The least-square mean (s.e.m.) change from baseline to week 24 derived from MMRM analysis was +0.10 (0.16) in the neflamapimod with C_trough_ >75th percentile (~5 ng/mL), compared to −0.13 (0.1) amongst placebo recipients (difference not significant) and −0.21 (0.11) in the neflamapimod subjects with C_trough_ <75th percentile.

For the HVLT-R, alternative versions were utilized in each session to minimize learning effects. However, this could not be done for the WMS, because there are no alternative versions. As a result, in the placebo group, there were significant learning effects, especially in subjects not on background AD therapy, resulting in significant improvements in WMS Immediate and Delayed Recall scores from baseline to week 24 (Fig. 3b). However, this learning effect was less evident in subjects on background AD therapy, presumably because they had more advanced disease. With that limitation, similarly to the relationship for the primary endpoint, neflamapimod subjects with higher C_trough_ levels appear to have less worsening in WMS Immediate and Delayed Recall, particularly the subjects on background AD therapy. In the prespecified analysis, no differences in the outcome on WMS Immediate and Recall were noted in neflamapimod subjects with C_trough_ >75th percentile compared with placebo recipients in the overall patient population (Supplemental Figure 2a). However, in an exploratory analysis, within those receiving background AD therapy, there were positive trends in the outcome on WMS Immediate and Delayed Recall, favoring neflamapimod subjects with C_trough_ >75th percentile compared with placebo recipients (Supplemental Figure 2b). In additional exploratory analyses, neflamapimod subjects with the highest C_trough_ drug levels also showed minor trends towards less worsening of both the MMSE and CDR-SB, compared with the placebo group (Supplemental Figure 3).

Table 3 shows the effects on CSF biomarkers by C_trough_ in neflamapimod subjects compared with placebo, utilizing the pre-specified cut-off of 75th percentile for C_trough_ in neflamapimod subjects. For T-tau, p-tau_181_ and Aβ_42_ a significant (*p*<0.05) plasma trough dependent effect was evident. Only minor or no trends were evident in the other 3 CSF biomarkers, perhaps due to the greater within-subject variability in change for these three biomarkers compared with the first three.

**Table 3:** Median (range) change from baseline to week 24 in plasma biomarkers by C_trough_ plasma neflamapimod levels on day 21 versus placebo

Biomarker	Placebo (***N*** = 67)	Neflamapimod by C_**trough**_		***P*** value for downward trend^**1**^
		≤ 75% Percentile (***N*** = 42)	> 75% Percentile (***N*** = 13)	
Total tau	7.0 (−84.2, 489.2)	2.9 (−68.4, 48.4)	3.6 (−30.7, 22.8)	0.04
P-tau_181_	0.67 (−6.4, 44.0)	0.03 (−5.5, 6.8)	0.06 (−4.2, 3.0)	0.02
Aβ_1-40_	221 (−6753, 4988)	465 (−6017, 8524)	126 (−2070, 2568)	0.24
Aβ_1-42_	−1.6 (−150, 268)	5.5 (−298, 205)	−37.7 (−136, 92)	0.05
Neurogranin	9.1 (−128, 481)	−0.65(−124, 323)	11.3 (−44, 75)	0.1
Neurofilament light chain	60 (−655, 3933)	42 (−1417, 513)	53 (−322, 665)	0.16

^1^Jonckheere-Terpstra test for downward trend of change from baseline for 3 groups: placebo, C_trough_ ≤75th percentile and C_trough_ >75th percentile

### Safety

Neflamapimod was well tolerated in this trial. Adverse events (AEs) occurring at ≥5% incidence in the neflamapimod group were fall (6% in neflamapimod, 4% in placebo), headache (6%, 4%), diarrhea (5%, 2%), and upper respiratory tract infection (5%, 8%). No on-study deaths were reported. Serious AEs (SAEs) reported in the neflamapimod group were hypokalemia and plasma cell myeloma (in different subjects), both considered unrelated to treatment. AEs leading to study drug discontinuation, each reported by 1 subject, included nausea and plasma cell myeloma (also an SAE) in the neflamapimod group and a fall leading to a subdural hematoma in the placebo group. Though analyses of group mean changes in alanine and aspartate aminotransferase levels did not reveal any differences between neflamapimod and placebo, one neflamapimod-treated subject had elevations in these enzymes to approximately 3 times the upper-limit-of-normal (ULN), which started to resolve within 1 week after treatment cessation, at which time the patient withdrew from the trial.

## Discussion

The major objective of this multi-center phase 2, randomized, double-blind placebo-controlled 24-week trial was to determine whether p38α inhibition could reverse synaptic dysfunction and, with it restore function, at least partially, as assessed by episodic memory performance. Toward that objective, in the primary endpoint, we found no difference between neflamapimod treatment and placebo in changes in episodic memory performance, as measured with the HVLT-R. However, the CSF biomarker results provide evidence suggesting that neflamapimod treatment had intended biological effects. In particular, significantly decreased levels of the disease biomarkers CSF T-tau and CSF p-tau_181_ were observed with neflamapimod, compared with placebo, over the 24-week treatment period in the full efficacy population. For both T-tau and p-tau_181_, there was a mean ~3% increase in CSF levels in the placebo group, as expected, while there was a decrease of similar magnitude in the neflamapimod group. Tau phosphorylation and tau pathology have been identified in preclinical studies as downstream consequences of abnormally high p38α kinase activity [10, 16, 20]. Further, neflamapimod treatment in the Ts2 transgenic mouse model decreased, relative to vehicle, levels of p-tau (pS202) in the brain (Nixon RA, personal communication; presentation at Alzheimer’s Disease Genetics Global Symposium, 22 September 2020). Thus, as the reduction in tau phosphorylation is a consequence of p38α inhibition, the decreases in p-tau and tau in CSF in the current study demonstrate, at a minimum, target engagement for neflamapimod with respect to p38α inhibition. In addition, p-tau, tau, and neurogranin are the protein markers generally considered to be most closely associated with synaptic dysfunction in AD [34–40], suggesting that neflamapimod has a beneficial effect on synaptic dysfunction.

Results of the pre-specified PK–PD analyses suggest that a major factor in not meeting the primary clinical objective, despite significant effects of neflamapimod treatment on CSF biomarker levels, is that the dose of 40 mg twice daily was too low. Specifically, for the primary endpoint, HVLT-R, there were trends towards improvement, relative to placebo, from baseline to week 24 in subjects with either C_trough_ ≥4 ng/mL or by the pre-specified cut-off for analysis of 75th percentile for C_trough_ within the study (5.4 ng/mL). This observation was supported by results in the secondary measure of episodic memory, WMS Immediate and Delayed Recall composites, in subjects on background AD therapy. Among these, neflamapimod-treated subjects having C_trough_ levels above the pre-specified 75th percentile demonstrated significantly better outcomes on WMS Immediate and Delayed at weeks 12 and 24, than did those on placebo. The 4 to 5 ng/mL threshold is consistent with current understanding of the mechanism-of-action and potency of neflamapimod. When the trial was designed, there were no potency data available for neflamapimod in AD-relevant pharmacology; instead, the dose level of 40 mg was chosen based on effective doses of neflamapimod in aged rats [18]. Recent mechanistic studies indicate that a major pharmacological target of neflamapimod is endolysosomal dysfunction associated with the protein Rab5 [41]. In addition, neflamapimod was shown to block Aβ oligomer-induced, as well prion-induced, dendritic spine loss in hippocampal neurons [42]. The in vitro potency, EC_50_, of neflamapimod for reversing Rab5+ endolysosomal dysfunction and for blocking Aβ-oligomer or prion-induced dendritic spine loss is 5–12.5 ng/mL. As brain concentrations of neflamapimod in preclinical studies are approximately two-fold higher than in the plasma, those potency concentrations are overlapping with predicted brain concentrations based on a plasma drug concentration of 4 to 5 ng/mL.

The trial was designed and powered with a hypothesis, based results in the preclinical models and in the two phase 2a clinical trials, that there would be substantial improvement from baseline in episodic memory function within the neflamapimod treatment group. The sample size in the current trial was based on the results of the phase 2a trials where, in both the 16-patient, 12-week trial (Study 302) that assessed episodic memory with the WMS and the 9-patient, 6-week trial (Study 303) that assessed episodic memory with the HVLT-R, the effect size for improvement from baseline exceeded 0.6. Based on those results, the sample size for the current study provided >80% statistical power for an effect size of 0.45 for the difference between neflamapimod treatment and placebo. Thus, with the decline of 0.15 from baseline to week 24 in the HVLT-R *Z*-score in the placebo-group in the current trial, an improvement from baseline to week 24 within the neflamapimod treatment group of at least 0.3 in the HVLT-R *A*-score of 0.3 was required to demonstrate a statistically significant effect on the primary endpoint, an effect not seen. This difference in outcome between the current trial and the two earlier phase 2a studies may be related to the two major differences between the current trial and phase 2a: (1) the patients in the earlier studies were less advanced in the disease and not receiving background AD therapy, and (2) the phase 2a studies included a higher dose of neflamapimod and achieved higher blood drug concentration levels. An impact of background therapy on the outcome is suggested by the neflamapimod participants not on background therapy with C_trough_ ≥ 75th percentile having an improvement from baseline of 0.45 *Z*-score on the primary endpoint (see Supplemental Figure 1). However, there were only five participants included in this analysis and, so, no conclusions can be drawn from it. With regard to the doses utilized in phase 2a, in Study 302 that utilized the WMS, nine participants received the same 40-mg BID dose as in the current study and seven received 125-mg BID (note: due to differences in excipient ratios utilized in the drug capsules, the 125 mg resulted in plasma drug levels, on average, only 50% higher than with 40-mg BID). Within Study 302, a statistically significant PK–PD relationship was established [27], a relationship indicating that plasma drug levels resulting from 125-mg BID were associated with greater improvement in WMS scores. In retrospect, and after obtaining the WMS results in the current study, the “improvement” seen in the 40 mg BID group likely resulted primarily from practice/learning effects, though the additional improvement at 50% higher plasma drug levels may still have been related to neflamapimod treatment. In the other phase 2a study (Study 303), in which positive effects on HVLT-R were demonstrated, the average plasma drug exposure with 40-mg BID was approximately 50% higher than in the current study because extremely low weight subjects were included. We believe that the results in Study 303 were not due to practice effects, as there were no practice/learning effects evident on the HVLT-R in the current study, including in the participants not receiving background therapy. Overall, though limited by the small number of participants in the phase 2a studies, the plasma drug concentration–effect relationships in phase 2a are generally consistent with the PK–PD analyses in the current study and suggest that the therapeutically active dose range is at least 50% higher than 40-mg BID (e.g., ≥ 40-mg TID).

With a specific kinase inhibitor, where both biomarker and clinical effects would depend on inhibition of that target kinase, the expectation would be that the dose–response would be similar for the biomarker and clinical effects. In this case, the differences between neflamapimod and placebo effects on the biomarkers were modest (~5%) which, in the absence of a clinical effect, suggests that the 40-mg BID dose level is simply at the lower end of the pharmacologically active dose range. Indeed, biomarkers are, by design, intended to be more sensitive than clinical effects and it is not unusual to see biomarker effects preceding clinical effects with respect to dose (i.e. at a lower dose) and/or time. For example, with aducanumab, significant effects on brain amyloid plaque by PET scan were seen at 3 mg/kg, though the clinical effects are limited to a dose of 10 mg/kg, where a moderately greater effect on amyloid plaque reduction is also seen [43]. Furthermore, at the clinically efficacious 10 mg/kg dose level, the majority of the effect on amyloid plaque load is seen by 26 weeks, while the clinical effect is not evident until week 52. To determine whether such relationships between biomarker and clinical effects exist for neflamapimod in AD will require further upward dose-ranging to first establish a clinical effect. In addition, a longer duration clinical trial may show a more distinct plasma drug concentration–effect relationship (i.e., dose–response), in terms of CSF biomarker effects, than we were able to demonstrate in the current study.

Non-CNS AEs, particularly aminotransferase elevations, have limited development of p38 MAPK inhibitors for peripheral inflammatory disorders [44]. Neflamapimod has the potential to minimize such toxicities, while maintaining robust pharmacological effects in the brain. The reasons for this include that plasma drug concentrations are half that in the brain and the drug is 95% protein-bound in whole blood, further reducing peripheral effects, as protein-binding decreases its potency three-fold. Our results support this concept, as only one of 78 neflamapimod recipients developed aminotransferase elevation to 3 times ULN, while pharmacological activity was demonstrated by the CSF biomarker results. Further, as the incidence of aminotransferase levels ≥ 3 times ULN was approximately 15% in a prior study of neflamapimod in rheumatoid arthritis patients, at a dose of 250 mg twice daily (C_trough_ approximately 30 ng/mL) [44], a low incidence of liver enzyme elevation is expected with dosing regimens that would consistently achieve C_trough_ ≥4 ng/mL.

### Limitations

This trial has limitations. First, the 24-week duration of the trial was not designed to ascertain effects on clinical disease progression. The sample size was effectively further attenuated because only a minority of subjects achieved plasma drug levels in the identified potentially therapeutically active range. In the PK–PD analysis, the sparse sampling approach utilized did not provide sufficient information to develop a robust population PK model, which would have allowed for a more thorough evaluation of the relationship between outcomes and PK parameters other than C_trough_. Our two measures of episodic memory each has its respective strengths and limitations. The WMS as a composite of three different cognitive tests provides the more comprehensive assessment of memory function. In addition, having three modestly correlated cognitive tests inherently decreases variability for the composite assessment. However, with the repeated application of the WMS, which has no alternative versions (that is, the same version is applied at each visit), over the relatively short-time period of the study, we saw substantial practice effects in treatment naïve patients that precluded any ability to discern neflamapimod effects. With the HVLT-R, which has alternative versions, at a group level, there were very little practice effects, as there was very little change in mean HVLT-R *Z*-scores over the 24 weeks of the study. However, with a single test, there was substantial within-subject variability from visit to visit. For example, within the placebo group, from baseline to week 6, nearly all scores for subjects above the median at baseline decreased, while those for all the subjects below the median increased; that is, there was substantial regression to mean. One approach to handling such variability would be to have more than one assessment at baseline, for example, one during the first screening visit and one on day 1 before starting treatment. The optimal approach would be to have a cognitive testing battery composed of three or more distinct episodic memory tests, each with alternate versions.

## Conclusions

This clinical trial did not show an effect of improving episodic memory function with 40-mg neflamapimod twice daily for 24 weeks in patients with mild AD. As the PK–PD analyses suggest that neflamapimod-treated subjects with higher plasma levels showed less episodic memory decline, an insufficient dose may have contributed to the lack of clinical efficacy. However, at this dose level, neflamapimod treatment modestly decreased CSF protein markers of synaptic dysfunction relative to placebo treatment. The results indicate that a longer duration clinical trial of neflamapimod at a higher dose level to assess effects on AD progression is warranted.

## Supplementary Information

**Additional file 1.**

**Additional file 2.**

**Additional file 3.**

## Data Availability

Clinical and biomarker endpoint datasets will be made available upon reasonable request to the sponsor, EIP Pharma.
