# Supplementary material for: A phase 2 double-blind placebo-controlled 24-week treatment clinical study of the p38 alpha kinase inhibitor neflamapimod in mild Alzheimer’s disease
Source: Alzheimers Res Ther. 2021 May 27;13:106. doi: 10.1186/s13195-021-00843-2 (PMC8157623; doi:10.1186/s13195-021-00843-2)
Supplement: Supplementary file 1 — Additional file 1. [file 13195_2021_843_MOESM1_ESM.pdf]

Supplemental Figure 1. Least score mean (s.e.m.) change in total z-score of HLVT-R total and delayed recall by C_{trough} for patients (A) not on background therapy, and (B) on background thearpy.

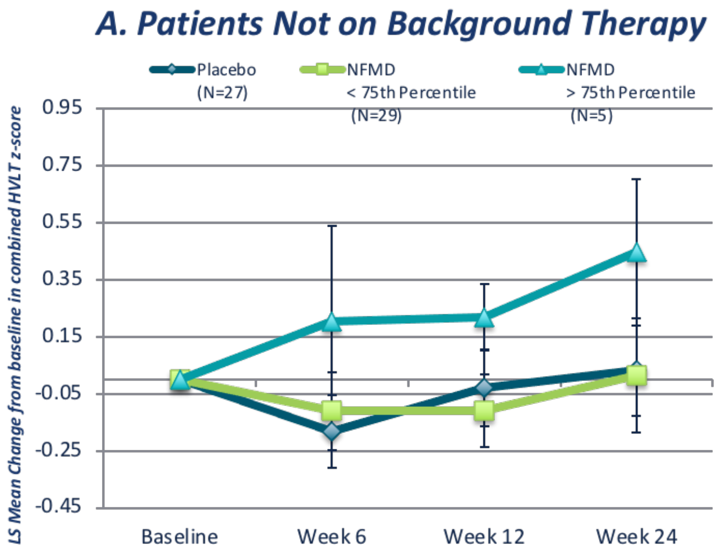

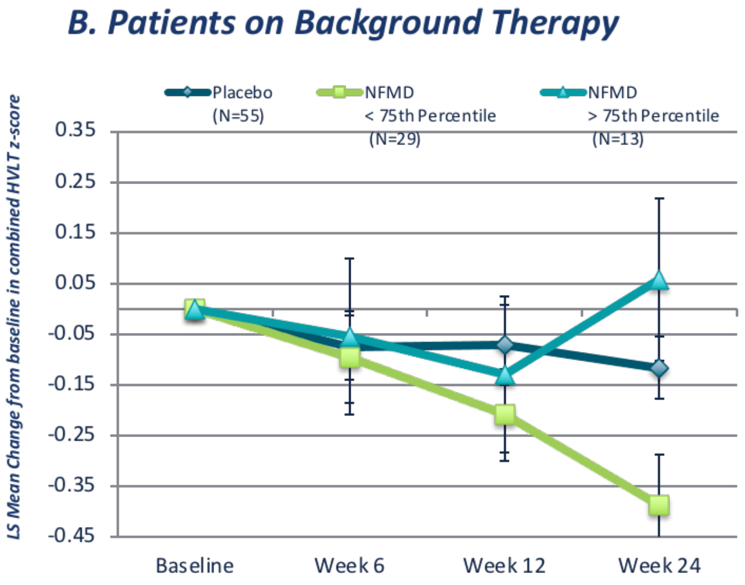

Note: Background therapy was either cholinesterase inhibitor (85% of participants) or memantine (15%)
