# Supplementary material for: A phase 2 double-blind placebo-controlled 24-week treatment clinical study of the p38 alpha kinase inhibitor neflamapimod in mild Alzheimer’s disease
Source: Alzheimers Res Ther. 2021 May 27;13:106. doi: 10.1186/s13195-021-00843-2 (PMC8157623; doi:10.1186/s13195-021-00843-2)
Supplement: Supplementary file 2 — Additional file 2. [file 13195_2021_843_MOESM2_ESM.pdf]

Supplemental Figure 2. Mean (s.e.m.) Change in WMS Combined Immediate and Delayed Recall by C_{trough} Levels

2a. Overall population

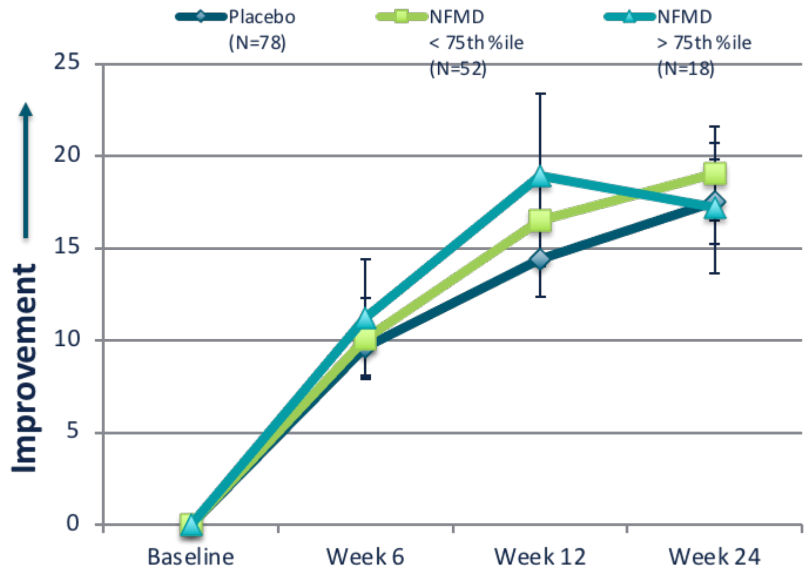

2b. Participants on background therapy

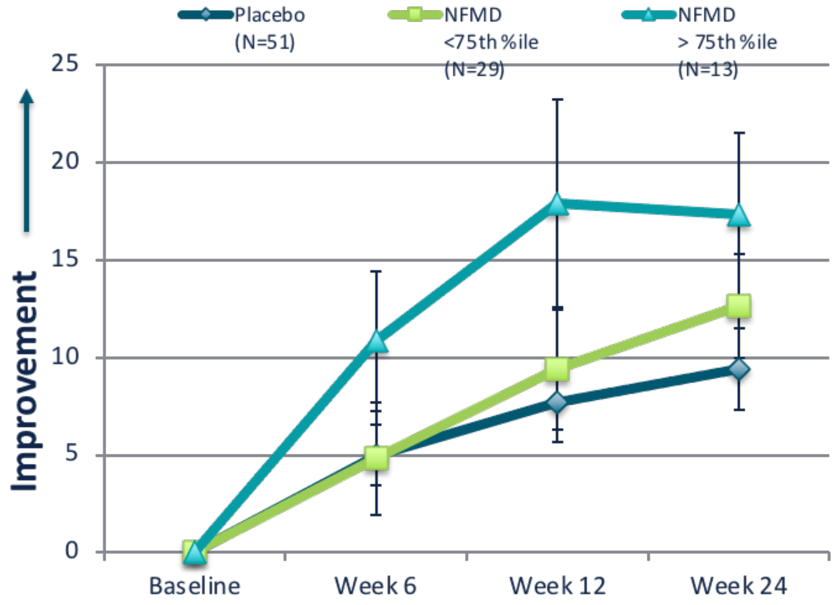

Note: Background therapy was either cholinesterase inhibitor (85% of participants) or memantine (15%)
