# Supplementary figures and images for: A phase 2 double-blind placebo-controlled 24-week treatment clinical study of the p38 alpha kinase inhibitor neflamapimod in mild Alzheimer’s disease
Source: Alzheimers Res Ther. 2021 May 27;13:106. doi: 10.1186/s13195-021-00843-2 (PMC8157623; doi:10.1186/s13195-021-00843-2)

## Supplemental Figure 2

### A. MMSE by $C_{\text{trough}}$

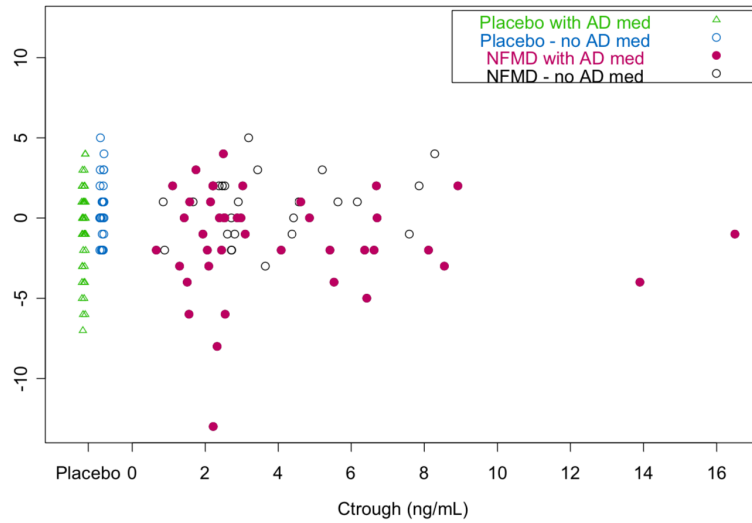

### B. CDR-SB by $C_{\text{trough}}$

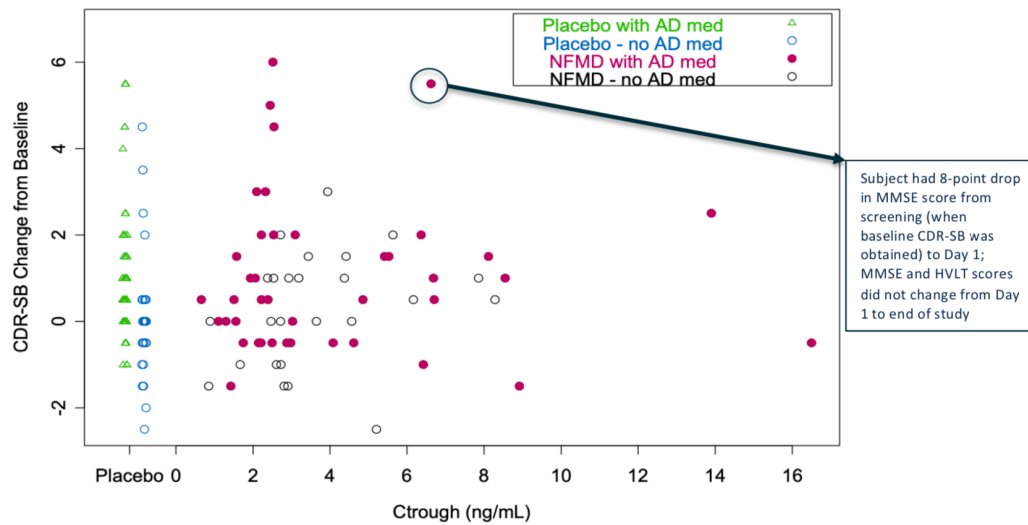

Supplement: Supplementary file 3 — Additional file 3. [file 13195_2021_843_MOESM3_ESM.pdf]
